# Phenylacetylglutamine as a novel biomarker of type 2 diabetes with distal symmetric polyneuropathy by metabolomics

**DOI:** 10.1007/s40618-022-01929-w

**Published:** 2022-10-25

**Authors:** J. Xu, M. Cai, Z. Wang, Q. Chen, X. Han, J. Tian, S. Jin, Z. Yan, Y. Li, B. Lu, H. Lu

**Affiliations:** 1 Department of Endocrinology, Shuguang Hospital Affiliated to Shanghai University of Traditional Chinese Medicine, Shanghai, 201203 China; 2 Department of Emergency, Shanghai University of Traditional Chinese Medicine, Shanghai, 201203 China; 3 Department of Endocrinology and Metabolism, Huashan Hospital, Fudan University, Shanghai, 200040 China

**Keywords:** Type 2 diabetes, Distal symmetric polyneuropathy, Metabolomics, Phenylacetylglutamine, Biomarker

## Abstract

**Purpose:**

Type 2 diabetes mellitus (T2DM) with distal symmetric polyneuropathy (DSPN) is a disease involving the nervous system caused by metabolic disorder, while the metabolic spectrum and key metabolites remain poorly defined.

**Methods:**

Plasma samples of 30 healthy controls, 30 T2DM patients, and 60 DSPN patients were subjected to nontargeted metabolomics. Potential biomarkers of DSPN were screened based on univariate and multivariate statistical analyses, ROC curve analysis, and logistic regression. Finally, another 22 patients with T2DM who developed DSPN after follow-up were selected for validation of the new biomarker based on target metabolomics.

**Results:**

Compared with the control group and the T2DM group, 6 metabolites showed differences in the DSPN group (*P* < 0.05; FDR < 0.1; VIP > 1) and a rising step trend was observed. Among them, phenylacetylglutamine (PAG) and sorbitol displayed an excellent discriminatory ability and associated with disease severity. The verification results demonstrated that when T2DM progressed to DSPN, the phenylacetylglutamine content increased significantly (*P* = 0.004).

**Conclusion:**

The discovered and verified endogenous metabolite PAG may be a novel potential biomarker of DSPN and involved in the disease pathogenesis.

**Supplementary Information:**

The online version contains supplementary material available at 10.1007/s40618-022-01929-w.

## Introduction

Distal symmetric polyneuropathy (DSPN), one of the most common complications in type 2 diabetes mellitus (T2DM), is defined as the presence of symptoms and/or signs of peripheral nerve dysfunction in patients with diabetes after the exclusion of other causes. It is pathologically characterized by the presence of progressive large and small nerve fibers loss. Larger nerve fiber function could be assessed by electrophysiological examination or recently reported whole plantar nerve conduction in the early phase of disease, whereas the small fibers by skin biopsy [1, 2]. Classic clinical symptoms include the bilateral symmetric numbness, pain, par-aesthesia, and so on [3]. A series of hazards caused by DSPN, such as anxiety, sleep impairment, and lower limb ulcers, can not only decrease the patients’ quality of life, but also further aggravate the risk of physical disabilities and mortality. However, compared with rapid growth in the prevalence of DSPN, significant studies on pathogenesis and particularly effective intervention are likely underpowered. A Lancet study showed that strict glycemic control in patients with T2DM was only associated with a 5–9% relative reduction in the risk of DSPN [4]. Although lifestyle interventions and comprehensive control of the abnormal metabolic state can prevent DSPN to some extent, it is still difficult to powerfully control the progression when a diagnosis of DSPN is confirmed [5]. The existing mechanistic studies indicated that high concentrations of sorbitol, accumulation of advanced glycation end products (AGEs), activation of protein kinase C (PKC) pathway, and elevated intracellular levels of reactive oxygen species (ROS) are closely interlinked with peripheral nerves injury. But so far, based on the above findings, nearly all the clinical therapeutic agents, such as aldose reductase inhibitors, AGE inhibitors, PKC inhibitors, and antioxidant lipoic, did not exhibit excellent rate of improvement in the pilot trials [6–9]. These suggest that identification of the core pathological process and key endogenous substance in DSPN awaits further new breakthrough.

The rapid development of high-throughput omics technologies certainly poses many unprecedented opportunities for studies of complex chronic diseases [10]. Since DSPN belongs to a chronic metabolic disease, metabolomics is well suited for a full assessment of it. The metabolomics allowed the comprehensive detection and quantification analysis of small molecule metabolites. Combined with appropriate data processing methods, the multitiered abnormal metabolic information can be obtained. Blood, being an important part of the internal environment, is an ideal medium for testing direct or indirect metabolic changes. Therefore, based on the above understandings, our study intends to explore the significant changes in metabolites and metabolic pathways of DSPN by blood metabolomics.

In this study, we first analyzed differences among the healthy controls, patients with T2DM and patients with T2DM associated DSPN by untargeted metabolomics. Additionally, the subgroup analysis of DSPN patients was performed. To identify the signature metabolites considered as potential biomarker in DSPN, we determined the overall screening principle: the significant change of metabolite concentrations should consistently coincide with disease progression. Which means they showed the same changing tendency not only among the above three groups but also in groups with different disease severity. After obtaining two potential biomarkers phenylacetylglutamine (PAG) and sorbitol, we examined the upstream and downstream metabolites changes and further explored abnormal metabolic pathway and sources of them. Finally, another independent population was selected for the validation of the new potential biomarker PAG by targeted metabolomics. More details are provided below.

## Subjects and methods

### Study design and participants

The study consisted of the screening cohort and the validation cohort. A total of 120 participants were enrolled in the screening cohort. Patients with T2DM (T2DM group, *n* = 30) and patients with T2DM associated DSPN (DSPN group, *n* = 60) were admitted to the Department of Endocrinology, Shuguang Hospital Affiliated to Shanghai University of Traditional Chinese Medicine, China, between June 2019 and January 2020. Healthy controls (control group, *n* = 30) who had recently completed physical examinations between March 2019 and September 2019 were recruited from Daqiao Community Health Service Center and Zhenru Community Health Service Center of Shanghai, China. The diagnostic criteria of T2DM were based on World Health Organization (1999) [11]. Details on the inclusion criteria of the DSPN patients in our study are provided in the section “Neuropathy assessment” below. The exclusion criteria included diabetic ketosis, ketoacidosis and severe infection tendency; patients with severe liver or kidney insufficiency (e.g., transaminases > 2.5 times the upper limit of normal or estimated glomerular filtration rate (e-GFR) < 60 mL/min/1.73 m^2^); pregnant or lactating women; intake of hormone drugs or drugs that may greatly affect metabolism; mental illness or cognitive disorders; other known causes of neuropathy (cervical/lumbar lesions, cerebral infarction, Guillain–Barre syndrome, and serious arteriovenous vascular disease).

The validation cohort comprised 22 patients with T2DM (but not the DSPN) who visited five community health centers in Jing’an District, China (Jing’an Temple, Shimen 2nd Road, Jiangning, Caojiadu, and Nanjing West Road Health Centre) between January 2014 and December 2019, and were subsequently all diagnosed with DSPN after an average follow-up period of five and a half years. All follow-up work was completed by the Endocrinology Department of Huashan Hospital. The diagnostic criteria of the diseases were as before. Since the biomarker discovered by the screening cohort is strongly associated with cardiovascular diseases (CVD) and involved in platelet aggregation, additional exclusion criteria were added in the validation cohort: abnormal coagulation function or haematological disorders; coronary heart disease or patients with major adverse cardiovascular events (such as myocardial infarction, stroke); taking adrenergic receptor inhibitors and anticoagulants within 3 months before the completion of follow-up. Each participant signed an informed consent before enrolment. The study protocol was approved by the ethics committee of Shuguang Hospital Affiliated to Shanghai University of Traditional Chinese Medicine (no. 2018-599-28-01).

### Neuropathy assessment

Distal symmetric polyneuropathy was defined according to the guidelines for prevention and treatment of type 2 diabetes in China (2017). Specific clinical criteria include: (1) patients with clear history of T2DM, (2) neuropathy ascertained during or after the diagnosis of T2DM, (3) the symptoms and signs of patients should be consistent with the performance of diabetic peripheral neuropathy, and (4) for symptomatic patients (numbness, pain, and paresthesia, etc.), any of the 5 examinations abnormality (ankle reflexes, pinprick sensation, vibration, pressure perception, and temperature sensation); for patients without symptoms, at least 2 of the 5 examinations were abnormal. In addition, all the patients were assessed overall using the Toronto Clinical Scoring System (TCSS) scores by the same experienced endocrinologist. Patients were included in our study only if they met both DSPN diagnostic criteria above and TCSS scores ≥ 6 [12]. TCSS is a scale for the diagnosis and evaluation for DSPN. The score ranges from 0 (absence of neuropathy) to 19 (severe neuropathy), 6 of which from symptoms assessment, 8 from lower limb tendon reflexes, and 5 from sensory examination of distal toes. DSPN patients also can be classified into mild (6–8 points), moderate (9–11 points), and severe (12–19 points) according to TCSS scores. The Achilles tendon and quadriceps tendon were examined by a reflex hammer to assess lower extremities deep tendon reflexes, a safe sterile needle for pain sensation testing (the volar aspect of the first, third, and fifth distal toes), Tip-Therm (Germany) for temperature sensation testing, 10 g monofilament for light touch sensation testing (one dorsal and nine plantar sites per foot), 128-Hz tuning fork was placed on the bony prominence of hallux for vibration sensation testing and repeated three times for at least one sham stimulation (incorrect answers more than once judged to be abnormal), and passive extension or flexion of the toes to test position sensation. Previous clinical research has confirmed that TCSS could effectively evaluate the presence and severity of DSPN compared to the gold standard of sural nerve biopsy [12].

### Clinical data and biochemical measurements

All participants should complete a standard questionnaire to document clinical data, including age, sex, the initial diagnosis date of diabetes, height, weight, smoking/alcohol use, and past medical history. Blood pressure was measured on the right upper arm with the participant after a 10 min resting interval. Venous blood samples were collected from all patients in the early morning for biochemical examination (fasting time was ≥ 8 h). Fasting plasma glucose (FPG), blood lipid, liver, and kidney functions were detected by an automatic biochemical analyser (AU680, Beckman Coulter, USA). Glycated Haemoglobin (HbA1c) was measured by high-performance liquid chromatography (Bio-Rad, Hercules, USA). After a standard breakfast (100-g steamed bread meal), 2-h postprandial C-peptide and plasma insulin levels were measured by immunological methods (Cobas E602, Roche, Germany). Urine albumin excretion was measured by a radioimmunoassay and the urinary albumin-to-creatinine ratio (ACR) was then calculated (AU5800, Beckman Coulter, USA).

### Blood sample collection and preparation for metabolomic

Fasting elbow venous blood samples from all 144 participants were collected in the early morning and put into the blood collection tube containing EDTA anticoagulant. After gentle mixing, the blood was centrifuged at 4000 RPM for 15 min at 4 °C, and then, the upper plasma was collected and immediately stored at -80 °C. Samples were prepared using the automated MicroLab STAR^®^ system from Hamilton Company. Several recovery standards were added prior to the first step in the extraction process for quality control purposes. To remove proteins, small molecules bound to proteins or trapped in the precipitated protein matrix were dissociated, and to recover chemically diverse metabolites, proteins were precipitated with methanol under vigorous shaking for 2 min (Glen Mills GenoGrinder 2000) followed by centrifugation. The resulting extract was divided into five fractions: two for analysis by two separate reverse phases (RP)/UPLC–MS/MS methods with positive-ion mode electrospray ionization (ESI), one for analysis by RP/UPLC–MS/MS with negative-ion mode ESI, one for analysis by hydrophilic interaction chromatography (HILIC)/UPLC–MS/MS with negative-ion mode ESI, and one sample was reserved for backup. Samples were placed briefly on a TurboVap^®^ (Zymark) to remove the organic solvent. The sample extracts were stored overnight under nitrogen before preparation for analysis. In addition, several types of controls were analyzed in concert with the experimental samples: a pooled matrix sample generated by taking a small volume of each experimental sample (or alternatively, use of a pool of well-characterized human plasma) served as a technical replicate throughout the data set; extracted water samples served as process blanks; and a cocktail of quality control standards that were carefully chosen not to interfere with the measurement of endogenous compounds was spiked into every analyzed sample, allowing instrument performance monitoring and aided chromatographic alignment. For detailed quality control operations and results, refer to the references and Supplementary Table 1.

### UPLC–MS/MS analysis

Untargeted metabolomic method was carried out using a Waters ACQUITY ultra-performance liquid chromatography (UPLC) and a Thermo Scientific QExactive high-resolution/accurate mass spectrometer (MS) interfaced with a heated electrospray ionization (HESI-II) source and Orbitrap mass analyzer operated at 35,000 mass resolution. The sample extract was dried and then reconstituted in solvents compatible to each of the four methods. Each reconstitution solvent contained a series of standards at fixed concentrations to ensure injection and chromatographic consistency. The extract was gradient eluted from a C18 column (Waters UPLC BEH C18-2.1 × 100 mm, 1.7 µm) and an HILIC column (Waters UPLC BEH Amide 2.1 × 150 mm, 1.7 µm). The MS analysis alternated between MS and data-dependent MSn scans using dynamic exclusion. The scan range varied slighted between methods, but covered 70–1000 *m*/*z*. After extracting the raw data, peak identified, normalization, and quality control processed. We obtained the retention time/index (RI), mass-to-charge ratio (*m*/*z*), and chromatographic data (including MS/MS spectral data) on all molecules. Finally, high-quality data set was made available for statistical analysis and data interpretation.

Targeted metabolomic approach with SCIEX TripleQuad 6500MD triple quadrupole liquid chromatography–tandem mass spectrometer was used. The PAG standard is shown in Supplementary Fig. S1. Data collection is based on Analyst MD 1.6.3 software, and Multi-Quant software was used for quantitative analysis.

### Statistical analysis

Univariate and multivariate statistical analyses of untargeted metabolomic results were conducted using SIMCA-P software (version 14.1, Umetrics, Umea, Sweden). In univariate analysis, the *P* value < 0.05 was considered as statistically significant. In addition, the Benjamini–Hochberg false discovery rate (FDR) procedure corrected the *P* values for multiple hypothesis testing. Multivariate analysis included Principal component analysis (PCA) and orthogonal partial least-squares discrimination analysis (OPLS-DA) analysis. *P* < 0.05, FDR < 0.1 [13], and variable importance in the projection (VIP) > 1 in the OPLS-DA model were set as the cut-off criteria for differential metabolites.

SPSS software (version 25.0) was used for general statistical analysis. A *t* test was conducted when the measurement data were conforming to the normal distribution and homogeneous variance, while Mann–Whitney *U* test was conducted when not conforming to the normal distribution. Chi-square test or non-parametric rank sum test was used for counting data. ROC curve analyses were used to estimate the optimal cut-off points of differential metabolites and assess the predictive performance. Binary logistic regression model was performed to further screen potential biomarkers based on cut-off points.

## Results

### Clinical characteristics of participants

**Fig. 1:**
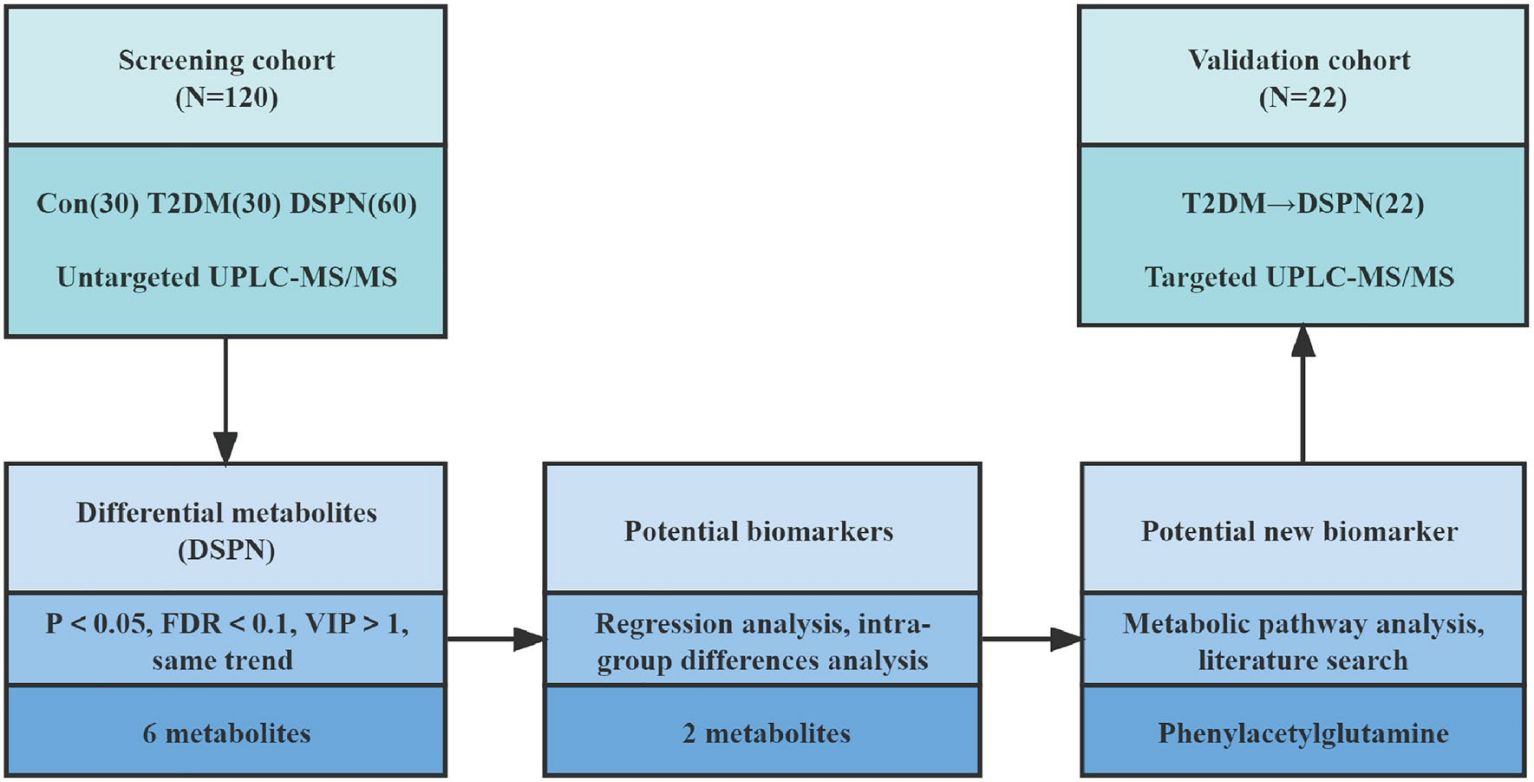
A brief flowchart of the study

The brief flowchart of the whole study is schematically illustrated in Fig. 1. The clinical characteristics of the all the participants are shown in Table 1. In the screening cohort, compared with the control group, patients in the DSPN group tended to be older (*P* = 0.104) and displayed higher levels of systolic blood pressure (SBP) (*P* = 0.012) and diastolic blood pressure (DBP) (*P* < 0.001). The levels of alanine aminotransferase (ALT) and high-density lipoprotein cholesterol (HDL-C) showed significant differences both in the T2DM and DSPN group compared to the control group. Compared with the T2DM group, patients in the DSPN group displayed longer diabetes duration (*P* = 0.033), larger proportions with CVD (*P* = 0.007), and lower level of e-GFR (0.009). There were no significant differences in the use of antidiabetic drugs, proportions of patients with diabetic retinopathy, glucose metabolism indicators, lipid metabolism indicators, and blood pressure between the T2DM group and the DSPN group, which represented that most baseline characteristics were comparable. We also found no significant differences in sex, smoking and drinking status, body mass index (BMI), triglyceride (TG), total cholesterol (TC), low-density lipoprotein cholesterol (HDL-C), and urine albumin/creatinine ratio (ACR) among the three groups.

**Table 1:** Clinical characteristics of all the participants

Characteristic	The screening cohort			The validation cohort	
	Control group (*N* = 30)	T2DM group (*N* = 30)	DSPN group (*N* = 60)	T2DM (*N* = 22, before follow-up)	DSPN (*N* = 22, after follow-up)
Age (years)	59.40 ± 9.95	55.30 ± 10.42	63.58 ± 8.40^##^	61.91 ± 5.01	67.36 ± 4.81
Sex (female/male)	16/14	12/18	31/29	12/10	12/10
Diabetes duration (years)	–	6.83 ± 4.66	8.18 ± 3.94^#^	5.05 ± 3.92	10.50 ± 4.24
Retinopathy (*N*, %)	–	3 (10.00)	9 (15.00)	2 (9.09)	5 (22.73)
History of CVD (*N*, %)	0 (0.00)	8 (26.67)	34 (56.67)^##^	–	–
OAD only (*N*, %)	–	22 (73.33)	43 (71.67)	15 (68.18)	16 (72.73)
Insulin only (*N*, %)	–	1 (3.33)	4 (6.67)	1 (4.55)	0 (0.00)
Insulin + OHA (*N*, %)	–	7 (23.33)	13 (21.67)	6 (27.27)	6 (27.27)
Smoking (*N*, %)	7 (23.33)	5 (16.67)	9 (15.00)	4 (18.18)	2 (9.09)
Alcohol intake (*N*, %)	7 (23.33)	2 (6.67)	6 (10.00)	4 (18.18)	4 (18.18)
BMI (kg/m^2^)	24.45 ± 2.21	24.83 ± 3.23	24.87 ± 3.46	25.52 ± 3.11	25.61 ± 2.94
TCSS score					
6–8	–	–	38(63.33)	–	–
9–11	–	–	15(25.00)	–	–
12–19	–	–	7(11.67)	–	–
SBP (mmHg)	121.63 ± 12.41	126.9 ± 12.45	128.97 ± 11.49*	135.27 ± 12.44	141.18 ± 18.25
DBP (mmHg)	74.37 ± 6.78	78.30 ± 7.32	78.93 ± 7.48**	82.73 ± 5.68	81.73 ± 8.20
FPG (mmol/L)	4.88 ± 0.36	7.12 ± 1.74	7.25 ± 1.79	9.09 ± 1.82	8.18 ± 2.73
FPG (mg/dL)	87.85 ± 0.36	128.17 ± 1.74***	130.51 ± 1.79***	163.64 ± 1.82	147.25 ± 2.73
HbA1c (%)	5.7 ± 0.2	7.8 ± 1.6***	7.8 ± 1.4***	7.5 ± 1.3	7.6 ± 1.2
C-peptide 0 min (ng/mL)	–	2.02 ± 1.08	1.97 ± 0.90	1.76 ± 1.04	0.84 ± 0.30^##^
C-peptide 120 min (ng/mL)	–	5.15 ± 3.09	4.48 ± 1.97	4.43 ± 2.86	2.48 ± 1.43
Insulin 0 min (mIU/mL)	–	10.77 ± 4.49	10.23 ± 6.12	12.62 ± 8.68	15.71 ± 11.91
Insulin 120 min (mIU/mL)	–	43.19 ± 20.03	39.93 ± 13.06	39.88 ± 27.31	49.01 ± 29.35
TG (mmol/L)	1.68 ± 0.98	1.46 ± 0.86	1.50 ± 0.88	1.85 ± 0.82	2.05 ± 1.08
TC (mmol/L)	5.10 ± 0.98	4.68 ± 0.81	4.67 ± 1.38	5.01 ± 0.78	4.96 ± 0.83
HDL (mmol/L)	1.40 ± 0.44	1.12 ± 0.30**	1.16 ± 0.31**	1.20 ± 0.20	1.31 ± 0.36
LDL (mmol/L)	2.97 ± 0.81	2.82 ± 0.79	2.74 ± 1.01	2.69 ± 0.89	2.74 ± 0.66
ALT (U/L)	17.33 ± 7.17	29.27 ± 16.58**	25.95 ± 12.93**	22.60 ± 11.64	26.23 ± 17.06
AST (U/L)	19.33 ± 4.64	21.43 ± 8.84	23.22 ± 9.47	21.74 ± 7.23	20.41 ± 6.55
Cr (ummol/L)	73.00 ± 14.80	61.00 ± 10.94**	63.04 ± 17.13**	64.82 ± 13.58	78.36 ± 23.40^#^
e-GFR (mL/min/1.73 m^2^)	97.70 ± 15.83	115.55 ± 24.12**	100.32 ± 24.27^##^	86.89 ± 15.80	79.05 ± 17.34^##^
ACR (mg/g)	11.89 ± 6.83	13.46 ± 9.07	13.45 ± 14.89	24.04 ± 21.45	36.07 ± 28.79^#^

Data are shown as n or the mean ± standard deviation

*CVD* cardiovascular disease, *OADs* oral antidiabetic drugs, *BMI* body mass index *SBP* systolic blood pressure, *DBP* diastolic blood pressure, *FPG* fasting plasma glucose, *HbA1c* glycated haemoglobin, *TG* triglyceride, *TC* total cholesterol, *HDL-C* high-density lipoprotein cholesterol, *LDL-C* low-density lipoprotein cholesterol, *ALT* alanine aminotransferase, *AST* aspartate aminotransferase, Scr serum creatinine, *e-GFR* estimated glomerular filtration rate, *ACR* urinary albumin-to-creatinine ratio, *TCSS* Toronto clinical scoring system

*P < 0.05, **P < 0.01, ***P < 0.001, compared to Control group

^#^*P* < 0.05, ^##^*P* < 0.01, ^###^*P* < 0.001, compared to T2DM group

The validation cohort consisted of 22 patients; 10 of them are male patients. There were significantly lower concentrations of fasting and 2 h postprandial C-peptide and higher levels of ACR in patients with DSPN after follow-up. There is no significant difference in other metabolic indicators before and after follow-up.

### Metabolic profiling of plasma

All the compounds were identified by comparison to library entries of purified standards or recurrent unknown entities and a total of 938 named biochemicals were obtained. Since we collected plasma samples, EDTA was measured in all samples. Additionally, we detected antihyperglycemic drugs, such as metformin, gliclazide, sitagliptin, and other components of various drugs in the blood samples of patients with T2DM. Given that the exogenous substances can pose influences on experimental results, we only included all the endogenous metabolites for subsequent data analysis [14]. Based on biological properties, the endogenous metabolites were categorized into seven super pathways: amino acid, carbohydrate, cofactors and vitamins, energy, lipid, nucleotide, and peptide. Then, the holistic metabolic profile was visualized with a heatmap (Supplementary Fig. S2). Over 80% of endogenous metabolites belonged to amino acid and lipid.

### Identification of differential metabolites in DSPN between three groups

**Fig. 2:**
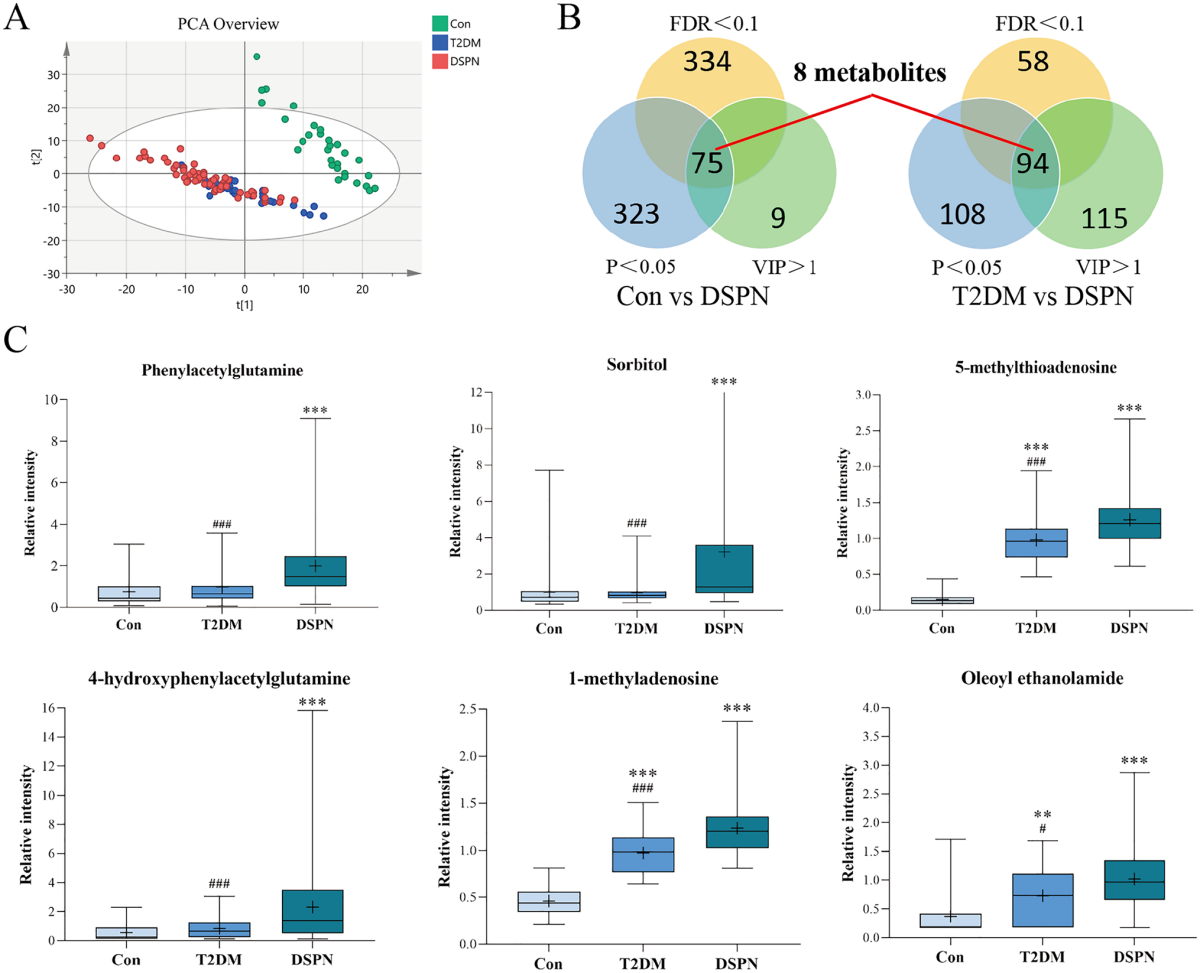
Identification of differential metabolites of DSPN. **a** A PCA plot between three groups. **b** Venn diagram of significant metabolites from the two pairwise comparisons. **c** Box plots diagram of six differential metabolites showing the same trend. The “+” in the figure represents means. The abscissa shows groups, and the ordinate represents relative intensity normalized by the peak value. *P < 0.05, **P < 0.01, and ***P < 0.001 when compared with control group, respectively; ^#^*P* < 0.05, ^##^*P* < 0.01, and ^###^*P* < 0.001 when compared with DSPN group

The differential metabolites obtained in the DSPN group should simultaneously meet the following conditions [15]: (1) in the comparison between the control group and the DSPN group, the T2DM group and the DSPN group, all metabolites should satisfy the criteria of VIP > 1, *P* < 0.05 and FDR < 0.1 (2) the metabolites showed the same trend in the comparison among the three groups. Here, we applied a relative more stringent criterion so as to identify specific differential metabolites associated with disease phenotype. PCA in multivariate data analysis showed that there was a significant difference in metabolite profile between the control group and the DSPN group and less difference between the T2DM group and the DSPN group (Fig. 2A). Permutation test revealed no overfitting situation in the two groups of models (Supplementary Figs. S3–4). In addition, VIP values obtained from the OPLS-DA models to assess the contributions of each metabolite. According to the screening criteria in (1) above, we obtained 75 different metabolites between the control group and the DSPN group, and 94 different metabolites between the T2DM group and the DSPN group, the heatmaps show different metabolites between groups (Supplementary Figs. S5–6). After taking the intersection, 8 metabolites were obtained (Fig. 1b), 6 of which showed an increasing trend among the three groups and were identified as differential metabolites of DSPN (Fig. 2c). They are 5-methylthioadenosine (MTA), 1-methyladenosine, 4-hydroxyphenylacetylglutamine, oleoyl ethanolamide, phenylacetylglutamine, and sorbitol (Supplementary Table 2).

### Further identification of potential biomarkers in DSPN

**Fig. 3:**
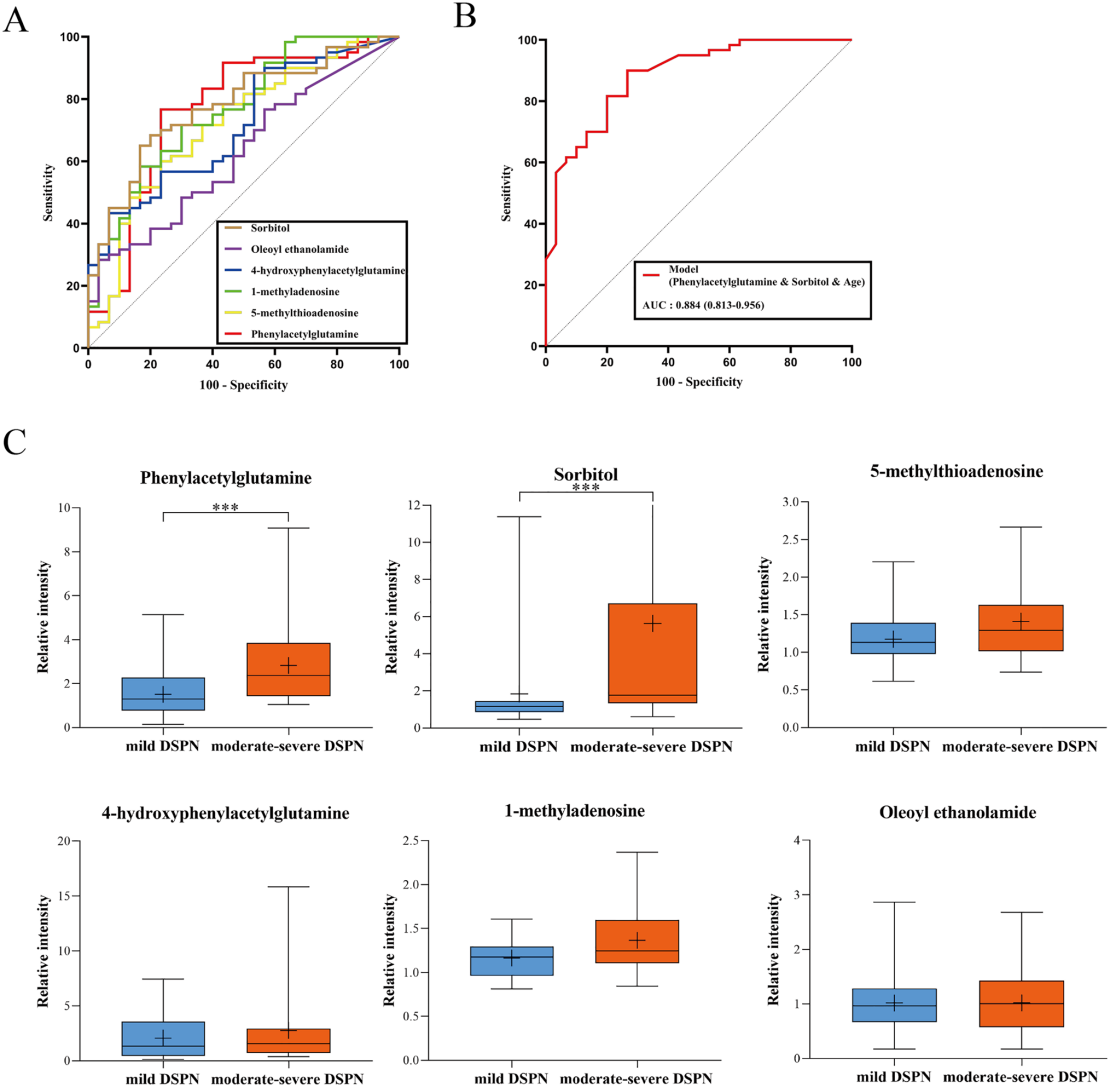
Identification of the potential biomarkers in DSPN. **a** ROC curve of each metabolite. **b** ROC curve for the final model. **c** Box plots for the comparison of six metabolites between the mild DSPN group and the moderate–severe DSPN group. ***P < 0.001

Potential biomarkers in DSPN were comprehensively screened using bivariate logistic regression analysis between T2DM and DSPN group as well as univariable analyses within the different DSPN severity groups. First, ROC curves of above 6 differential metabolites were performed to assess the optimal threshold values through the Youden index (Fig. 3a and Table 2), so the metabolites were transformed into categorical variables according to threshold values. Taking the disease as the dependent variable, entering 6 categorical variables into binary logistic regression analysis (forward: LR) to construct the optimal model, control variables included age, sex, diabetes duration, history of CVD, Retinopathy, FPG, HbA1c, ACR, and e-GFR (Age, diabetes duration, CVD, and e-GFR were adjusted because of the differences in baseline characteristics; sex, retinopathy, FPG, HbA1c, and ACR were additionally adjusted owing to the close association with the DSPN). Only the PAG (OR 3.61; 95% CI [confidence interval] 1.14–11.42), sorbitol (OR 6.89; 95% CI 2.08–22.77), and age (OR 1.03; 95% CI 1.02–1.18) appear in the model. Then, the ROC curve was displayed based on the probabilistic predictive values of above model equation, under which the area was 0.884 (95% CI 0.813–0.956). Compared with single metabolite, the model showed better discriminatory ability (Fig. 3b). Similarly, we ascertained phenylacetylglutamine and sorbitol as two independent risk factors for DSPN.

**Table 2:** Detailed information obtained from each metabolite ROC curve and identified optimal cut-off value

Variables	AUC	SE	95% CI	Maximum Youden index	Optimal threshold values	Sensitivity	Specificity
5-Methylthioadenosine	0.718	0.058	0.604–0.832	0.367	1.121	0.600	0.767
1-Methyladenosine	0.766	0.053	0.663–0.870	0.417	1.090	0.717	0.700
4-Hydroxyphenylacetyl glutamine	0.715	0.055	0.607–0.823	0.366	1.887	0.433	0.933
Phenylacetylglutamine	0.765	0.058	0.652–0.878	0.534	0.960	0.767	0.767
Oleoyl ethanolamide	0.627	0.060	0.509–0.745	0.250	1.294	0.283	0.967
Sorbitol	0.778	0.050	0.680–0.875	0.483	1.077	0.683	0.800

*AUC* area under the curve, *SE* standard error, *CI* confident interval

We further divided the 60 participants with DSPN into subgroups according to TCSS scores [16]. Because of the small number of severe DSPN patients, we integrated the last two groups and categorized patients into mild DSPN (*n* = 38) and moderate–severe DSPN (*n* = 22). The distributions of 6 differential metabolites between above two groups are shown as box plots (Fig. 3c). Again, only PAG (*P* < 0.001) and sorbitol (*P* < 0.001) exhibited significant difference between the groups. Through the above-mentioned comprehensive statistical analysis, we identified phenylacetylglutamine and sorbitol as potential biomarkers of DSPN. They were not only well related to the clinical phenotypes, but also significantly associated with the occurrence and development of DSPN.

### Metabolic pathway analysis of potential biomarkers

**Fig. 4:**
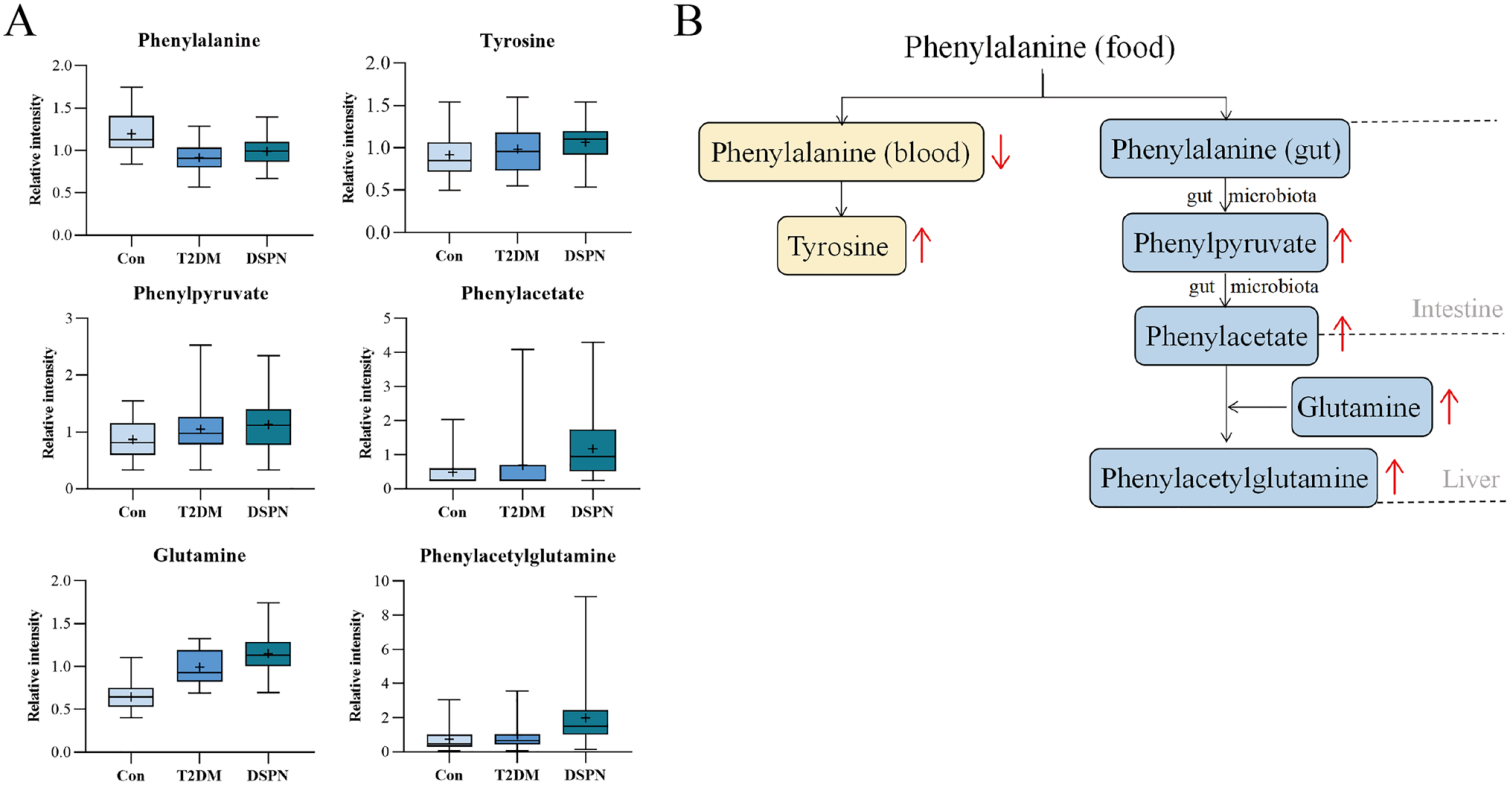
Details for PAG metabolism pathway. **a** The relative content difference of mapped 6 metabolites in the pathway by box plots. **b** The pathway flowchart of PAG metabolism. Red arrows indicate changes in metabolite content in the DSPN group compared with the Con/T2DM groups. Gray fonts within dotted lines indicate metabolism sites

The invivo metabolic pathways of 2 potential biomarkers were determined by the query of literature and relevant signaling pathways. Meanwhile, we were inspired by backtracking changes of upstream and downstream metabolites along the pathways. The complete metabolic pathway of PAG was detailed reported in the cell in 2020 [17]. PAG is initially derived from phenylalanine (Phe) in food. Most of the Phe will be absorbed from the small intestine directly into the blood and involved in tyrosin biosynthesis. Unabsorbed Phe could be oxidized and metabolized by gut microbiota to produce phenylacetate and enter the body blood circulation through the portal vein. Phenylacetate could combine with glutamine (Glu) to generate PAG in the liver (Fig. 4b) [17, 18]. Looking across above pathways, we found that the upstream substances of PAG, phenylacetate, and phenylpyruvate were showed a trend of upregulation in the DSPN group. This suggested that the change of gut microbiota was likely to be a main reason for higher PAG content in DSPN patients. In addition, a relatively decreasing blood Phe content in the T2DM and DSPN groups suggested that more Phe might be metabolized by gut microbiota (Fig. 4a).

**Fig. 5:**
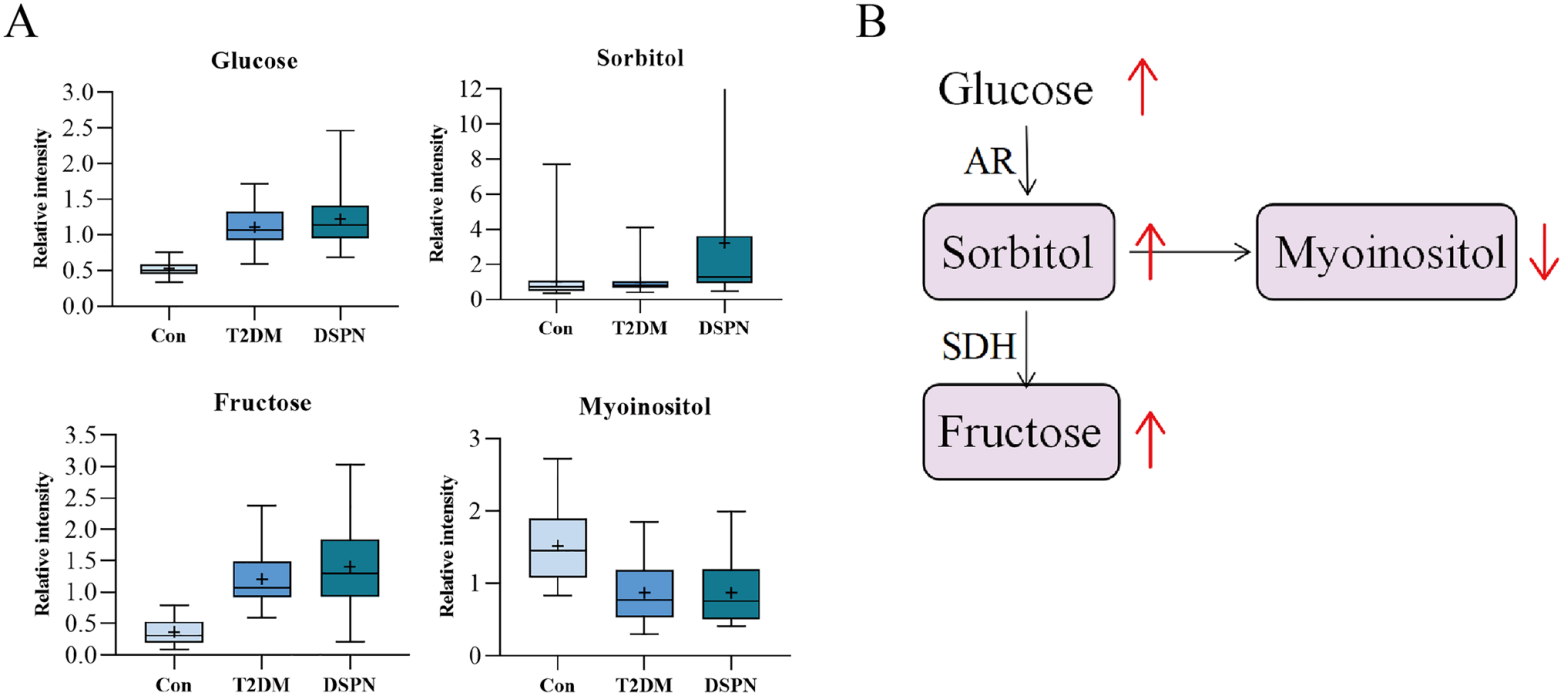
Details for Sorbitol metabolism pathway. **a** The relative content difference of mapped 4 metabolites in the pathway by box plots. **b** The pathway flowchart of sorbitol metabolism. Red arrows indicate changes in metabolite content in the DSPN group compared with the Con/T2DM groups. *AR* aldose reductase, *SDH* sorbitol dehydrogenase

Pathway related to sorbitol has already been confirmed to be associated with DSPN (Fig. 5b). Under neuropathy state, activation of the polyol pathway results in the accumulation of sorbitol and fructose, elevating intracellular osmolarity and causing inositol depletion [19]. Our metabolomic data also showed same changes in above metabolites (Fig. 5a), which indirectly confirms the accuracy of our data.

### Preliminary validation of the novel potential biomarker PAG

**Fig. 6:**
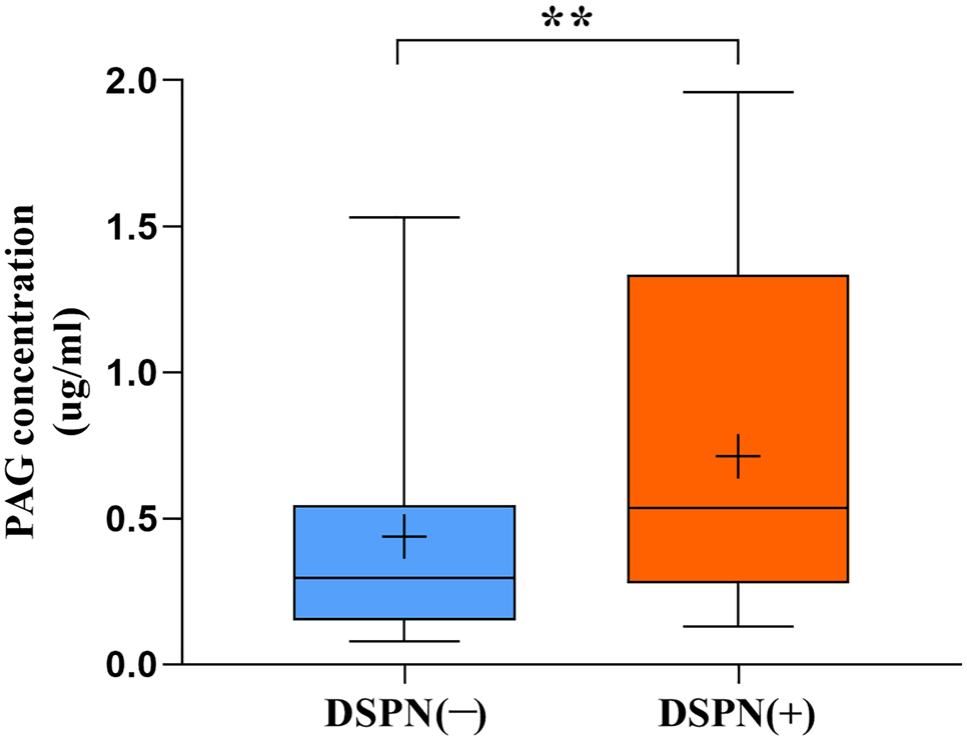
Box plot of PAG content change in the validation cohort. **P < 0.01

Considering that massive and intact studies have revealed the mechanism of sorbitol in DSPN, we speculated that PAG might be a new biomarker in DSPN, and further targeted validation was performed in a small cohort. Targeted metabolomics results showed that blood PAG concentrations were significantly increased after the patients with T2DM-only developed DSPN (*P* = 0.004) (Fig. 6).

## Discussion

To the best of our knowledge, this is the first research related to potential biomarkers in DSPN based on the combination of untargeted metabolomics and targeted metabolomics approach. Unlike the conventional metabolomics data analysis approaches, we identified sorbitol and phenylacetylglutamine as biomarkers of DSPN by screening the results layer by layer with a more stringent criterion. As previously explained, they changed significantly with the disease phenotypes. Also, the assessment of the metabolic pathways in the holistic context demonstrated the comprehensiveness and reliability of our data. Finally, quantitative targeted metabolomics study further proved the stability of PAG as a novel potential biomarker.

At present, there are only two metabolomics studies of diabetic polyneuropathy in humans being reported and have a reference value for our results. One study paid attention to the obesity patients with peripheral neuropathy (PN), and they found that compared with obesity patients without PN, the content of spermidine (considered as neuroprotective compound) significantly decreased, whereas the content of complex lipids, such as ceramides, lactosylceramides, and sphingomyelins, decreased [20]. Our research results exhibit a similar trend (Supplementary Fig. S7). Another metabolomics study found that 3-carboxy-4-methyl-5-propyl-2-furanpropanoate (CMPF) and phatidylcholines (PEs): phatidylcholines (PCs) ratio were closely associated with T2DDPN patients [21]. This trend could also be observed in our results (Supplementary Fig. S7). However, an opposite trend with citrate variation was observed in our study. Our results showed higher content of citrate and lower content of isocitrate and α-ketoglutarate located downstream, which suggested impaired TCA cycle flow and decreased aconitase activity (Supplementary Fig. S7). This phenomenon was confirmed in the animal model of peripheral neuropathy [22]. Furthermore, previous study demonstrated that citrate accumulation may induce insulin resistance [23]. The results we obtained contrary to the literature may be related to the overall glucose and lipid metabolism status or medication difference of clinical patients. Of additional concern, the levels of three compounds (CMPF, isoursodeoxycholate, and 3-formylindole) metabolized by human intestinal flora were significantly higher in DPN patients. The results are consistent with our metabolomics data and indicating a possible link between DPN and the intestinal flora.

In what follows, we focused on the obtained meaningful metabolites in our results. A total of six differential metabolites were found in the three comparison groups. Except for two metabolites further identified as potential biomarkers and discussed in detail below, another four differential metabolites also showed suggestive significance. Among them, 5-methylthioadenosine (MTA) was significantly higher in DSPN patients. MTA is a byproduct of polyamine synthesis and can suppress polyamine accumulation, such as spermine and spermidine (the changes of two metabolites are consistent with our results) [24]. It was found that elevated concentrations of MTA were observed in the diabetes state [25]. However, the potential neuroprotective effect of MTA has also been reported recently [26]. This may reflect a self-protective mechanism in the neuro-pathic state and warrant subsequent studies. 1-Methyladenosine is a modified nucleoside that was reported to be associated with an increased risk of death in T2DM [27]. Besides, it may be present due to neuronal oxidative stress injury [28]. 4-Hydroxyphenylacetylglutamine was identified only by metabolomics and might be related to tyrosine metabolism, but there is a lack of specific mechanism studies [29]. Oleoyl ethanolamide (OEA) is associated with endocannabinoid metabolism. Several studies reported that it could exert anti-inflammatory, antioxidant, and neuroprotective effects by activating PPAR-α [30–32]. Another study found that plasma OEA was increased in Alzheimer’s disease patients and suggesting a neuroprotective action of endocannabinoids in neurodegenerative disease [33]. This could help in better understanding our results. The above analysis results suggested possible links between the four differential metabolites and DSPN. However, this may be worth exploring in future work.

What interested us most were two potential metabolites PAG and sorbitol. Sorbitol has been proved to be critical for neural cell degeneration in the pathogenesis of DSPN. Abundant studies have revealed that excessive accumulation of sorbitol in nerve cells increases osmotic pressure of nerve cells and eventually leads to nerve oedema, degeneration, and necrosis [34]. In this study, we also obtained the iconic metabolite sorbitol, which was surprising and proved the reliability of our data to a certain extent. However, the clinical efficacy of targeted drugs, such as aldose reductase inhibitor, is not very satisfactory [6]. Therefore, we chose PAG as a research object and validated it by a longitudinal follow-up study. Likewise, the results showed that the change in blood PAG content is closely related to the disease status and might be a novel potential new biomarker of DSPN. Regrettably, we were unable to further evaluate whether PAG is a suitable biomarker for early prediction of DSPN due to the lack of data on patients who did not progress to DSPN in longitudinal study. Additionally, larger sample size studies are needed to extrapolate whether PAG can be used as a promising biomarker for disease diagnosis or monitoring.

**Fig. 7:**
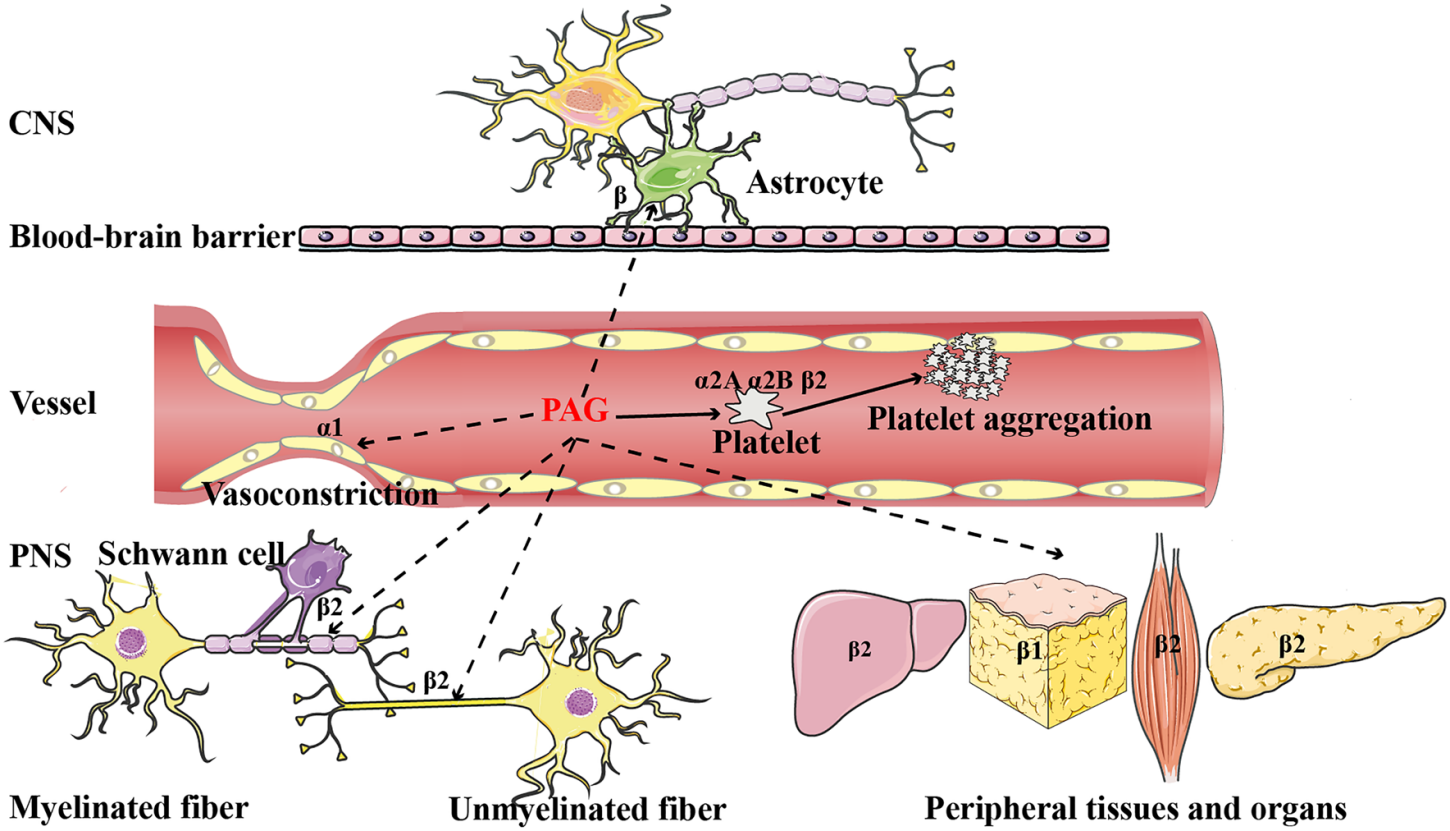
Diagram of the possible potential mechanism of PAG in DSPN state. Solid lines represent confirmed mechanisms, and dotted lines represent putative mechanisms. CNS, central nervous system. PNS, peripheral nervous system. α1, α2A, α2B, β, β1, β2, adrenergic receptor subtype

So far, several studies have reported that PAG showed different levels of content during various disease states [35–37], but a strong correlation between PAG and DSPN phenotype has never been reported. As outlined previously, Unabsorbed Phe could be oxidized and metabolized by *Clostridium sporogenes* in the intestinal flora and involved in PAG synthesis [18, 38]. The chemical structure of PAG is similar to catecholamine and can enhance platelet adhesion to collagen matrix by activating adrenergic receptors (ADRS)-α2A, α2B, and β2 on platelets as well as promote the increase of intracellular Ca^2+^ concentration to regulate thrombosis in vivo [17]. Interestingly, a study reported last year found that the richness of Firmicutes was remarkably elevated in the diabetic peripheral neuropathy group when compared with the T2DM group and healthy controls. While *C. sporogenes*, an essential participant in the regulation of PAG metabolism, belonged to Firmicutes. The study also indirectly confirmed the value of our results [39]. Significantly, a recent study proved the dose-dependent neurotoxicity of PAG in multiple sclerosis, and PAG could slow down the firing rate and conduction velocity of neurons independently of mitochondrial function and oxidative stress response [40]. In addition, above reference article also proved two other bacterial neurotoxic metabolites *p*-cresol sulfate and indoxyl sulfate. Totally like PAG, p-cresol sulfate undergoes *C. sporogenes* metabolism. Our results are still in keeping with above findings (Supplementary Fig. S8). After a thorough analysis of its structure, functions, and metabolic pathways by consulting extensive related literatures, we speculated that PAG is likely be involved in the pathogenesis of DSPN and the relationship between DSPN and PAG might exist as follows (Fig. 7).

1. DSPN is one of the most common diabetic microvascular complications, quite a few studies have reported that metabolic imbalance, microvascular damage, micro-circulation disorder, and abnormal hemorheology might play an important role in the occurrence and development of DSPN [41]. Peripheral nerves would undergo demyelination changes or axonal degeneration when micro-circulation is impaired, since peripheral nerves are mainly nourished by micro-vessels to maintain normal function. Meanwhile, increased vascular resistance can also lead to degeneration and necrosis of the corresponding nerves due to ischemia and hypoxia [42]. Platelet function was also an important factor affecting micro-vasodilation and hemorheology. The increase of vasoactive factors released by platelets might damage nerve function by affecting blood supply to micro-vessels [43]. E Williams observed the presence of platelet thrombus or even tiny infarcts in micro-vessels of nerve biopsy from 11 DSPN patients under electron microscope, which might be an important cause of peripheral nerve function damage in mice [44]. A study from the Lancet also illustrated increased platelet sensitivity and activation in DSPN patients [45]. PAG could enhance the reaction and aggregation of platelets to promote thrombosis, so PAG might participate in the occurrence and development of DSPN by affecting peripheral nerve and microvascular circulation. In addition, whether PAG has a direct action on vascular endothelial α1 receptors to cause vasoconstriction and blood supply to nerve tissues requires further experimental research and exploration.
2. Studies have found that activating adrenergic receptors (ADRs) were also present in the myelin sheath, plasma membrane, Schwann cells, and astrocytes of peripheral or central nervous tissues in mammals. The activation of β-ADRs on the surface might affect the nerve conduction of axons and the release of neurotransmitters [46, 47]. Although PAG was able to permeate through the blood–brain barrier, additional studies are needed to determine whether PAG can reach peripheric tissues and whether it could affect nerve conduction at appropriate concentrations.
3. The activation of ADRs in peripheral organs and tissues, such as the liver, pancreas, and adipose tissue, also has a profound impact on metabolism. Prolonged activation of the adrenaline system could lead to insulin resistance, glucose, and fatty acid metabolism disorders and mitochondrial dysfunction [48, 49]. Experimental studies have shown that the activation of ADRs could reduce insulin secretion by affecting mitochondrial function and inhibiting oxidative metabolism of islet cells; at the same time, it could affect glucagon secretion [50, 51]. Therefore, we speculate that PAG can extensively act on ADRs in peripheral tissues and promote the further development of the above-mentioned metabolic disorders leading to the occurrence and development of DSPN.

The primary limitation of our study was the relatively small sample number of validation cohort. However, the pre–post-comparison analysis based on longitudinal follow-up and reduced inter-individual variability might mitigate this limitation to some extent. Additionally, the lack of adjustment for therapies may affect circulating metabolites and related results. In the future, well-designed studies with large sample sizes and detailed clinical information should be undertaken to explore the specific role of PAG.

## Conclusion

In summary, based on the metabolomics technology, we identified the potential biomarker PAG in patients with DSPN for the first time through an experimental approach with 120 participants and a small validation population. PAG levels were significantly increased in the plasma of patients with DSPN and had a good power in discriminating between patients with DSPN and T2DM. Furthermore, it was closely correlated with DSPN severity. As a metabolite produced from intestinal microbiota, the discovery of PAG in DSPN brought two important reflections: whether the alteration of intestinal microbiota could contribute to the development of DSPN and whether PAG could play a vital role in the pathomechanism of DSPN.

## Supplementary Information

Below is the link to the electronic supplementary material.

Supplementary file1 (DOCX 5582 KB)

## Data Availability

Some or all datasets generated during and/or analyzed during the current study are not publicly available, but are available from the corresponding author on reasonable request (xujiahui95826@163.com).
