# Supplementary material for: Phenylacetylglutamine as a novel biomarker of type 2 diabetes with distal symmetric polyneuropathy by metabolomics
Source: J Endocrinol Invest. 2022 Oct 25;46(5):869–82. doi: 10.1007/s40618-022-01929-w (PMC10105673; doi:10.1007/s40618-022-01929-w)
Supplement: Supplementary file 1 — Supplementary file1 (DOCX 5582 KB) [file 40618_2022_1929_MOESM1_ESM.docx]

***Journal of Endocrinological Investigation***

**Supplementary Material**

Instrument variability was determined by calculating the median relative standard deviation (RSD) for the internal standards that were added to each sample prior to injection into the mass spectrometers. Overall process variability was determined by calculating the median RSD for all endogenous metabolites (i.e., non-instrument standards) present in 100% of the Matrix samples, which are technical replicates of pooled samples. Overall process variability was determined by calculating the median RSD for all endogenous metabolites present in the CMTRX (pool created by taking a small aliquot from every sample) technical replicates. Values for instrument and process variability meet our acceptance criteria as shown in the Supplementary Table 1 below.

**Supplementary Table 1** Data quality: instrument and process variability.

| QC Sample | Measurement | Median RSD |
| --- | --- | --- |
| Internal Standards | Instrument Variability | 4% |
| Endogenous Biochemicals | Total Process Variability | 7% |

*QC* quality control, *RSD* relative standard deviation.

**Supplementary Fig. S1** A PAG standard curve was plotted. Abscissa: concentration of PAG. Ordinate: peak area ratio. Calibration curves for PAG: y = 4.67376x + 7.77524e-6 (r = 0.999981).


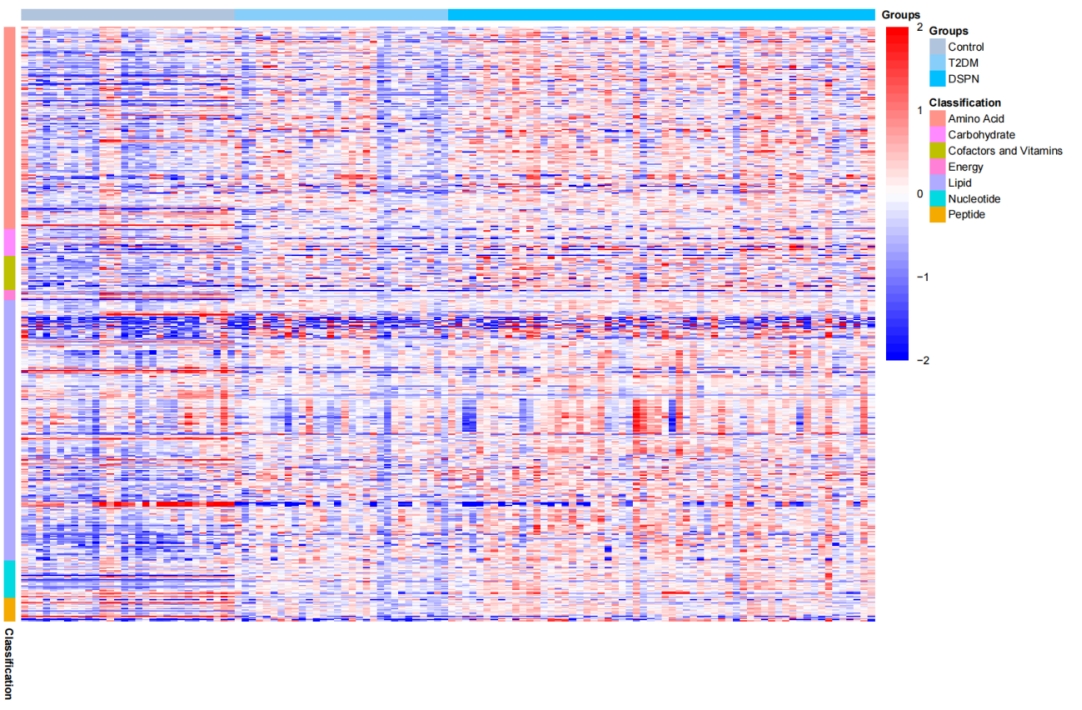


**Supplementary Fig. S2** Heatmap analysis of the relative distributions of all identified endogenous metabolites among the three groups. Relative abundance values of metabolites were scaled by natural logarithm (ln), ln (normalized value). The top of the graph indicates samples grouping. The left part lists seven super pathways of endogenous metabolites.


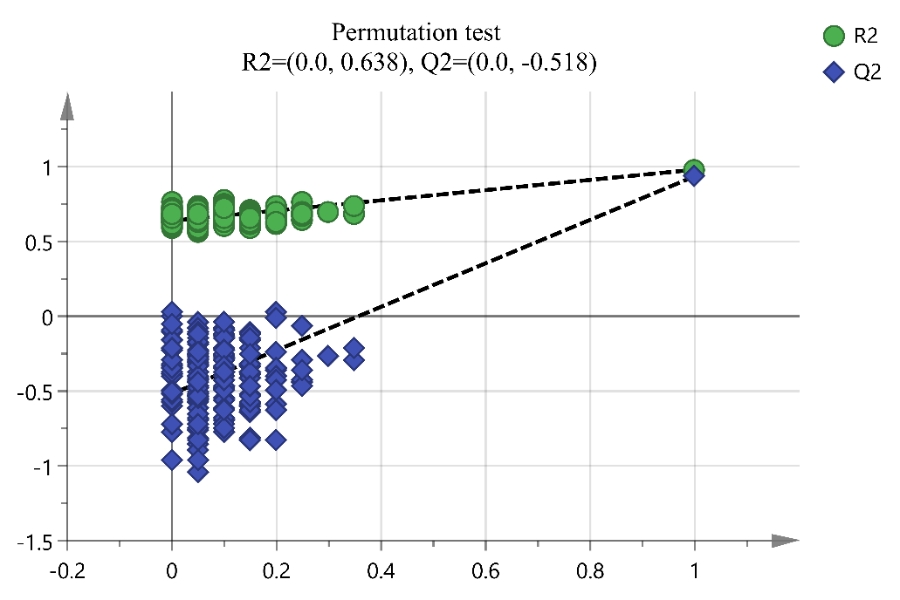


**Supplementary Fig. S3** Statistical validation of OPLS-DA model by permutation testing (200 iterations) from control group and DSPN group. The intercepts of R2 = (0.0, 0.638), Q2 = (0.0, -0.518) illustrate the OPLS-DA model is not over-fitting.


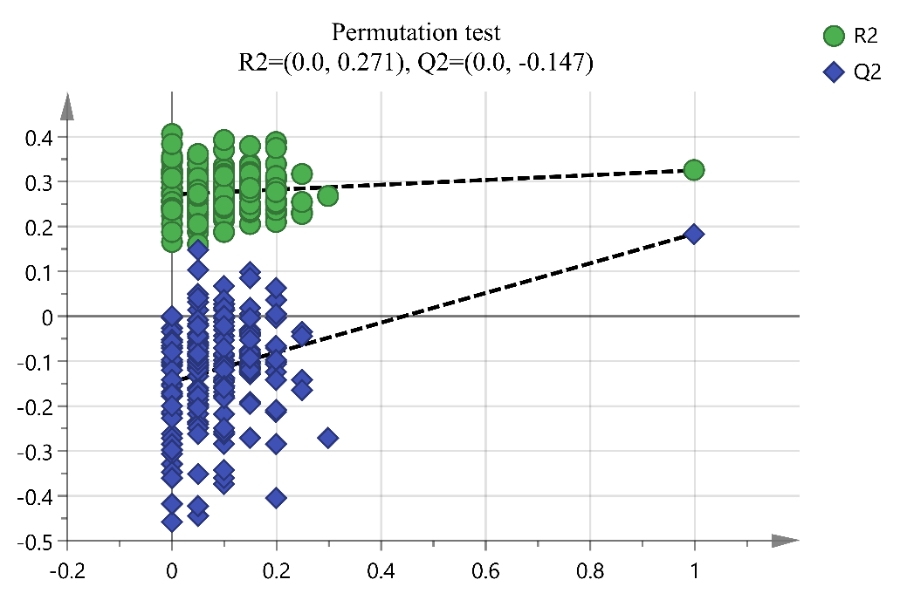


**Supplementary Fig. S4** Statistical validation of OPLS-DA model by permutation testing (200 iterations) from T2DM group and DSPN group. The intercepts of R2 = (0.0, 0.271), Q2 = (0.0, -0.147) illustrate the OPLS-DA model is not over-fitting.


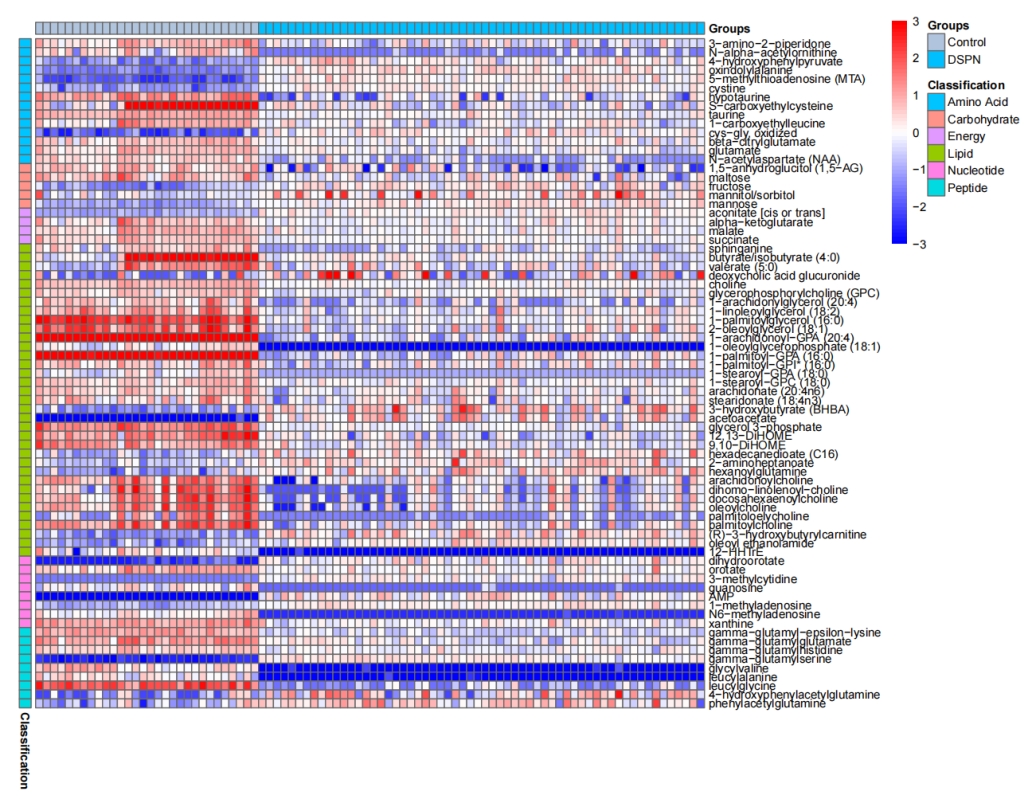


**Supplementary Fig. S5** Heatmap of 75 differential metabolites from the Control and DSPN groups. Relative abundance values of metabolites were scaled by natural logarithm (ln), ln (normalized value). The names of the corresponding metabolites are shown on the right side.


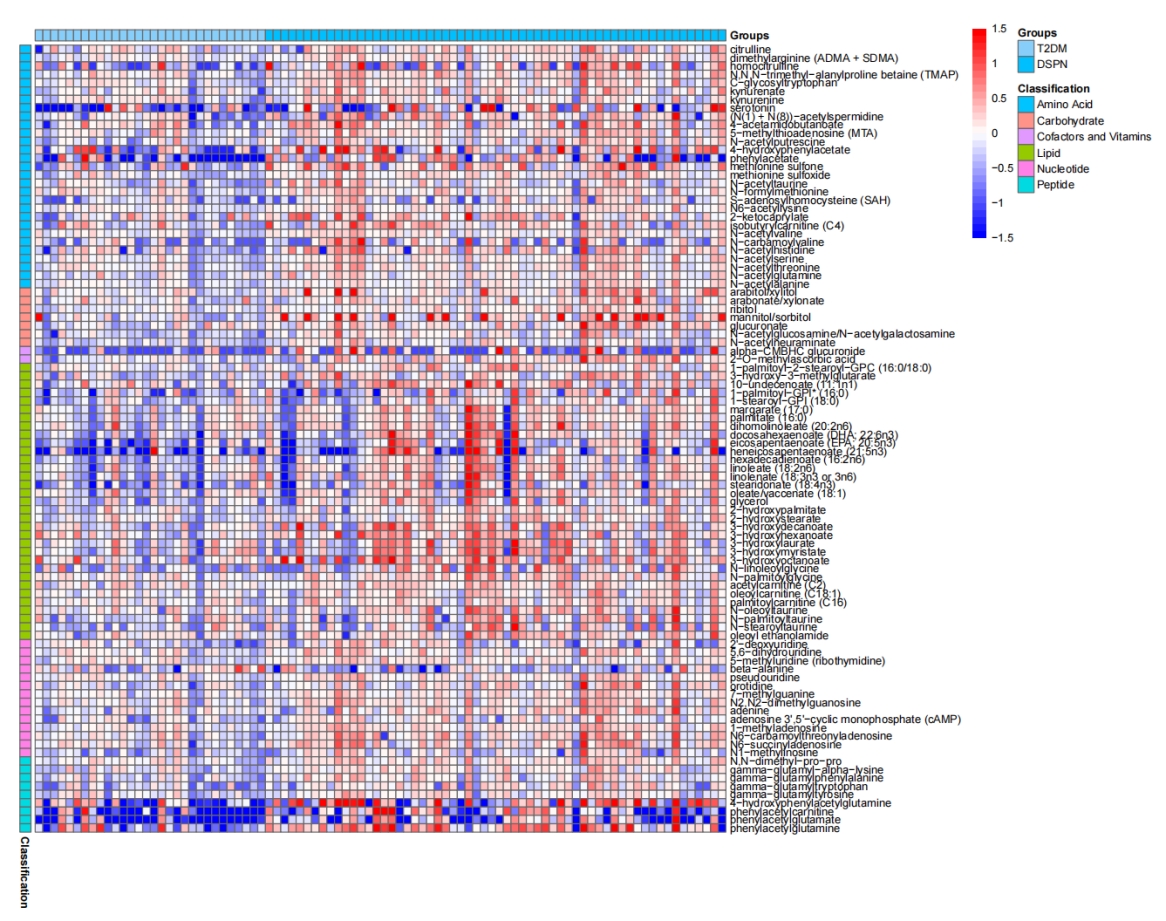


**Supplementary Fig. S6** Heatmap of 94 differential metabolites from the T2DM and DSPN groups. Relative abundance values of metabolites were scaled by natural logarithm (ln), ln (normalized value). The names of the corresponding metabolites are shown on the right side.

**Supplementary Table 2** Identified six differential metabolites in DSPN among three groups. The basic information annotated in LC-MS.

| **Metabolites** | **Control vs DSPN** | | | **T2DM vs DSPN** | | | **Super Pathway** | **Sub Pathway** | **MASS** | **RI** | **CAS number** | **Platform** |
| --- | --- | --- | --- | --- | --- | --- | --- | --- | --- | --- | --- | --- |
|  | **P value** | **FDR** | **VIP** | **P value** | **FDR** | **VIP** |  |  |  |  |  |  |
| **5-methylthioadenosine** | 9.63E-24 | 3.76E-22 | 1.20 | 0.00275 | 0.038319 | 1.50 | Amino acid | Polyamine metabolism | 298.10 | 2752 | 2457-80-9 | LC/MS positive |
| **1-methyladenosine** | 3.16E-22 | 1.08E-20 | 1.01 | 7.06E-05 | 0.019203 | 1.41 | Nucleotide | Purine metabolism, adenine containing | 282.12 | 2120 | 15763-06-1 | LC/MS positive |
| **4-hydroxyphenylacetylglutamine** | 0.00048 | 0.00093 | 1.13 | 0.00353 | 0.043628 | 2.42 | Peptide | Acetylated peptides | 279.10 | 1650 | — | LC/MS negative |
| **Phenylacetylglutamine** | 5.89E-05 | 0.000139 | 1.01 | 0.001451 | 0.03036 | 2.02 | Peptide | Acetylated peptides | 263.10 | 2330 | 28047-15-6 | LC/MS negative |
| **Oleoyl ethanolamide** | 1.25E-12 | 1.14E-11 | 1.02 | 0.016079 | 0.077408 | 1.29 | Carbohydrate | Endocannabinoid | 322.28 | 6150 | 68171-52-8 | LC/MS negative |
| **Sorbitol** | 0.02621 | 0.037555 | 1.13 | 0.022363 | 0.090786 | 2.42 | Carbohydrate | Fructose, mannose and galactose metabolism | 181.07 | 2320 | — | LC/MS polar |

*FDR* false discovery rate, *VIP* variable importance in the projection, *RI* retention index obtained in experimental conditions, *CAS* chemical Abstracts Service.


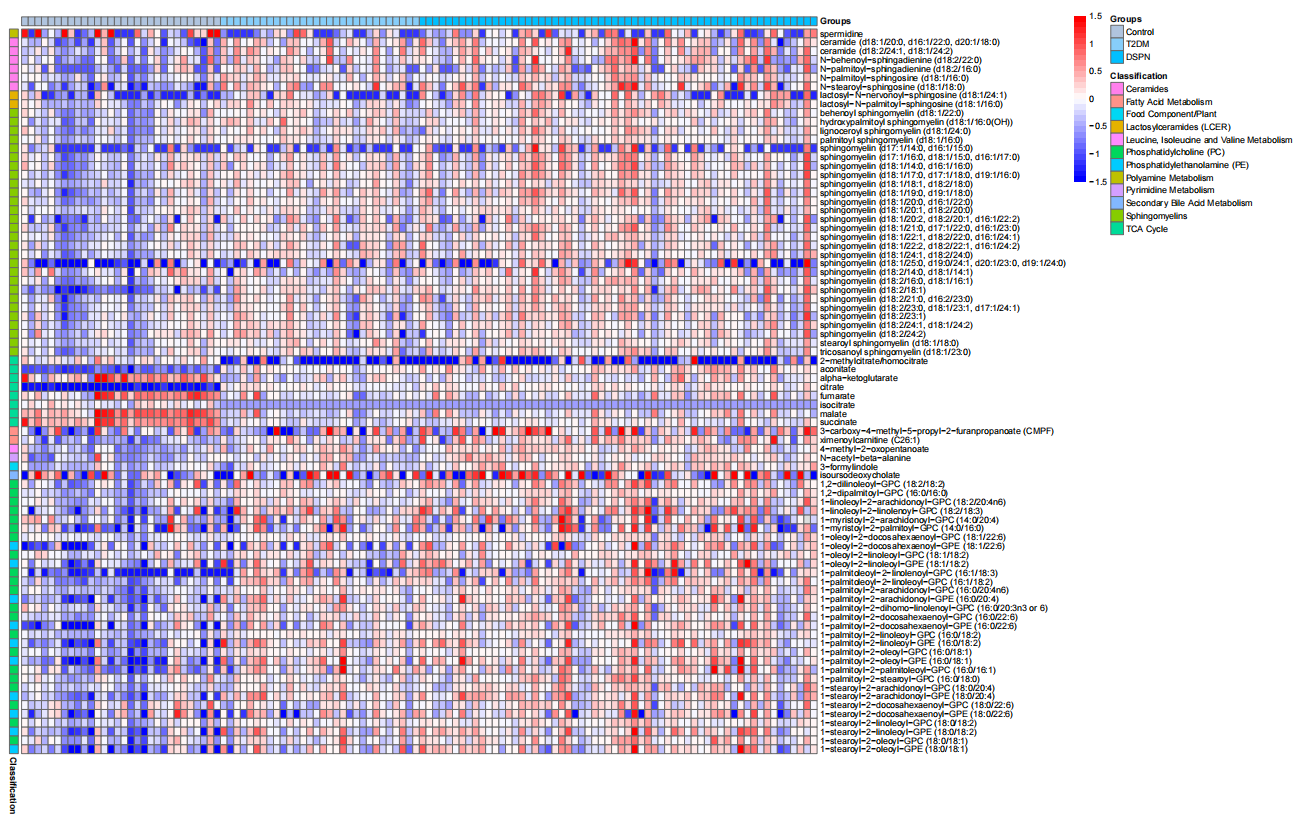


**Supplementary Fig. S7** Heatmap of total differential metabolites from two articles mentioned in the text (reference [17] and [18]). Relative abundance values of metabolites were scaled by natural logarithm (ln), ln (normalized value). Classification represents a sub-pathway of each metabolite.


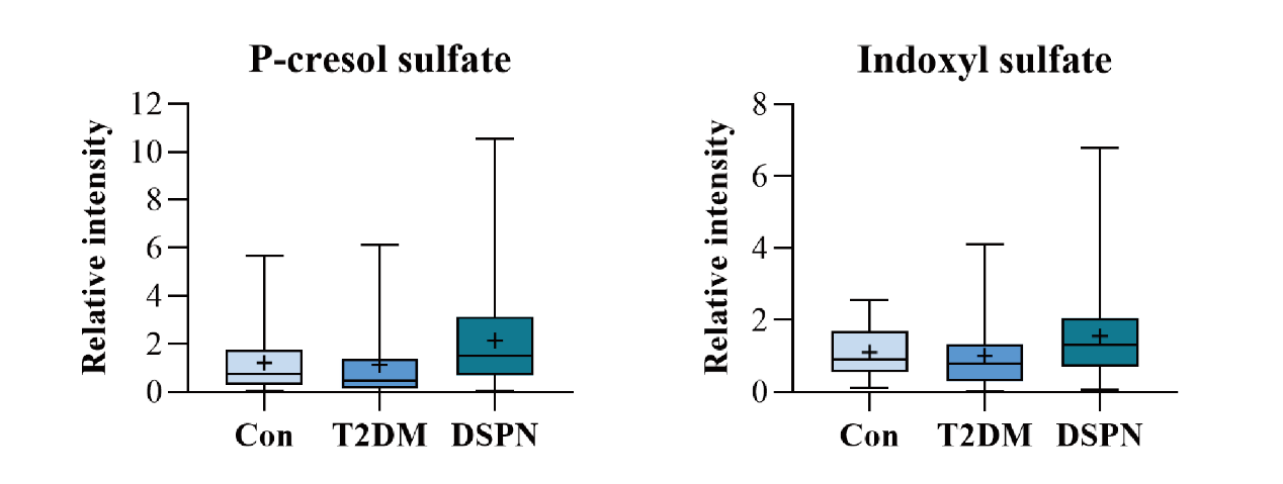


**Supplementary Fig. S8** Box plots diagram of two bacterial neurotoxic metabolites. The “+” in the figure represents means. The abscissa shows groups, the ordinate represents relative intensity normalized by the peak value.
